# Epigenetic Regulation Mediated by Methylation in the Pathogenesis and Precision Medicine of Rheumatoid Arthritis

**DOI:** 10.3389/fgene.2020.00811

**Published:** 2020-08-04

**Authors:** Shicheng Guo, Lingxia Xu, Cen Chang, Runrun Zhang, Yehua Jin, Dongyi He

**Affiliations:** ^1^Department of Medical Genetics, School of Medicine and Public Health, University of Wisconsin-Madison, Madison, WI, United States; ^2^Center for Precision Medicine Research, Marshfield Clinic Research Institute, Marshfield, WI, United States; ^3^Department of Rheumatology, Guanghua Hospital, Shanghai University of Traditional Chinese Medicine, Shanghai, China; ^4^Institute of Arthritis Research in Integrative Medicine, Shanghai Academy of Traditional Chinese Medicine, Shanghai, China

**Keywords:** epigenetic, methylation, rheumatoid arthritis, pathogenesis, regulation

## Abstract

Rheumatoid arthritis (RA) is a complex disease triggered by the interaction between genetics and the environment, especially through the shared epitope (SE) and cell surface calreticulin (CSC) theory. However, the available evidence shows that genetic diversity and environmental exposure cannot explain all the clinical characteristics and heterogeneity of RA. In contrast, recent studies demonstrate that epigenetics play important roles in the pathogenesis of RA, especially DNA methylation and histone modification. DNA methylation and histone methylation are involved in innate and adaptive immune cell differentiation and migration, proliferation, apoptosis, and mesenchymal characteristics of fibroblast-like synoviocytes (FLS). Epigenetic-mediated regulation of immune-related genes and inflammation pathways explains the dynamic expression network of RA. In this review, we summarize the comprehensive evidence to show that methylation of DNA and histones is significantly involved in the pathogenesis of RA and could be applied as a promising biomarker in the disease progression and drug-response prediction. We also explain the advantages and challenges of the current epigenetics research in RA. In summary, epigenetic modules provide a possible interface through which genetic and environmental risk factors connect to contribute to the susceptibility and pathogenesis of RA. Additionally, epigenetic regulators provide promising drug targets to develop novel therapeutic drugs for RA. Finally, DNA methylation and histone modifications could be important features for providing a better RA subtype identification to accelerate personalized treatment and precision medicine.

## Introduction

Rheumatoid arthritis (RA) is an autoimmune disease characterized by synovial hyperplasia and joint destruction (Lee et al., 2017). Its onset is progressive and invasive, which can lead to joint deformity and disability. In the past decades, linkage analysis and genome-wide association studies (GWAS) have identified >100 susceptibility genes, especially shared epitopes (SE), in *HLA* genes (Okada et al., 2019). Cell surface calreticulin (CSC), mediated by *PTPN22* and *PADI4*, has successfully been used to explain the onset of RA (Okada et al., 2014). However, the pathogenesis of RA is still not fully understood. Furthermore, the majority of the current RA drugs have not been developed against GWAS targets but cytokine and inflammatory pathways. RA-associated genetics thoroughly explain neither the heterogeneity of the clinical characteristics nor the treatment differences among patients with RA. In recent years, more and more studies have started to focus on the role of epigenetics in RA (Ai et al., 2018; Guo et al., 2019, 2020; Tseng et al., 2019a) and investigate the contribution of epigenetics to the heterogeneity of RA. DNA methylation and histone modifications, important epigenetic modifications that affect the expression of immune-related genes and inflammation progression (Meng et al., 2015; Lawrence et al., 2016) have become promising mechanisms to explain the pathogenesis of RA (Mazzone et al., 2019). Numerous studies have found that methylation in immune cells may lead to RA progression through coordinated control of immune cell differentiation and function (Qiu et al., 2017; Meng et al., 2019). In this review, we systematically summarize the progress of methylation research (DNA and histone) to enhance the understanding of RA pathogenesis. We also summarize the pieces of evidence that show methylation as an interface to connect genetic and environmental exposures and as a promising biomarker for diagnosis, treatment, and subtype identification. Finally, we show that epigenetic modules are promising novel biomarkers and drug targets for the next-generation personalized treatment and precision medicine.

## Genome-Wide Methylation Profiling to Identify RA-Associated Epigenetic Variants

DNA methylation is an important epigenetic modification that is involved in the regulation of gene expression and transcript splicing. Genome-wide identification of abnormal DNA methylation variations in FLS, innate and adopted immune cells, including B cells and T cells, provide a full spectrum of epigenetic pattern changes during the onset and progression of RA. Abnormal DNA methylation can be found at a very early stage of RA. Compared with normal synovial fibroblasts (SF), evidence shows that the CpG island located in the promoter region of *PM20D1*, *EN1*, and *SHROOM1* are hypermethylated in very early RA-derived synovial fibroblasts (veRASF). *MFAP2*, *RIMBP2*, *IRX6*, *DDR1*, and *HLA-C* are found hypermethylated in established, long-standing RA-SF (estRASF). DNA methylation profiles in cadherin, integrin, and Wnt cell adhesion signaling pathways; actin cytoskeleton components; and antigen presentation pathways notably change in veRASF and estRASF (Karouzakis et al., 2018). Compared with early RA (ERA), the global DNA methylation level is lower in the cell migration, differentiation, and adhesion pathways in long-standing RA (LRA), which may contribute to cell proliferation, differentiation, migration, and transition to chronic RA (Ai et al., 2015). DNA methylation changes in these pathways show a similar pattern with human cancers, implicating that synovial hyperplasia and invasion may be a shared underlying mechanism with human cancer metastasis (Wang et al., 2015). Moreover, extracellular matrix (ECM), cholesterol biosynthesis, and immune system pathways are also significantly enriched in RA high-risk individuals, indicating that DNA methylation signals may be useful in early diagnosis or risk evaluation of RA (Karouzakis et al., 2019).

Genome-wide methylation change was also found in T and B lymphocytes (Glossop et al., 2013, 2014; Rhead et al., 2017; Guderud et al., 2020). A study identified 150 and 113 CpG loci with unique methylation characteristics in T and B lymphocytes in patients with ERA (Glossop et al., 2016). Evidence shows that *ARSB* and *DUSP22* are hypermethylated and *GALNT9* and *MGMT* are found hypomethylated in the T lymphocytes (Glossop et al., 2014). Interestingly, *DUSP22* codes for a protein tyrosine phosphatase that negatively regulates STAT3 and IL-6/STAT3 signaling pathways, indicating DNA methylation-mediated *DUSP22* silencing, might be a fundamental effect to activate STAT signaling in RA (Sekine et al., 2006). *BARX2*, *ASB1*, *ADAMTS17*, and *MGMT* are found hypomethylated in the B lymphocytes and can be used to distinguish patients with RA from healthy individuals (Glossop et al., 2014).

Through genome-wide methylation analysis, the discovery of shared methylated regions and pathways in multiple immune diseases may suggest the existence of the same pathogenesis. A recent study identified 337 differential methylated genes shared between RA and Parkinson’s Disease (PD), which provides new evidence for the shared biological mechanism between RA and PD (Tang et al., 2018). Another study found that mitochondrial L-carnitine shuttle and PTEN signaling pathways are simultaneously differentially expressed in RA, systemic sclerosis (SSc), and systemic lupus erythematosus (SLE) (Hudson et al., 2017). In other recent studies, genome-wide DNA methylation profiles revealed common epigenetic patterns of interferon-related genes in multiple autoimmune diseases, including Graves’ disease (GD), RA, SLE, and SSc (Guo et al., 2017; Ding et al., 2018; Chen et al., 2019).

**FIGURE 1:**
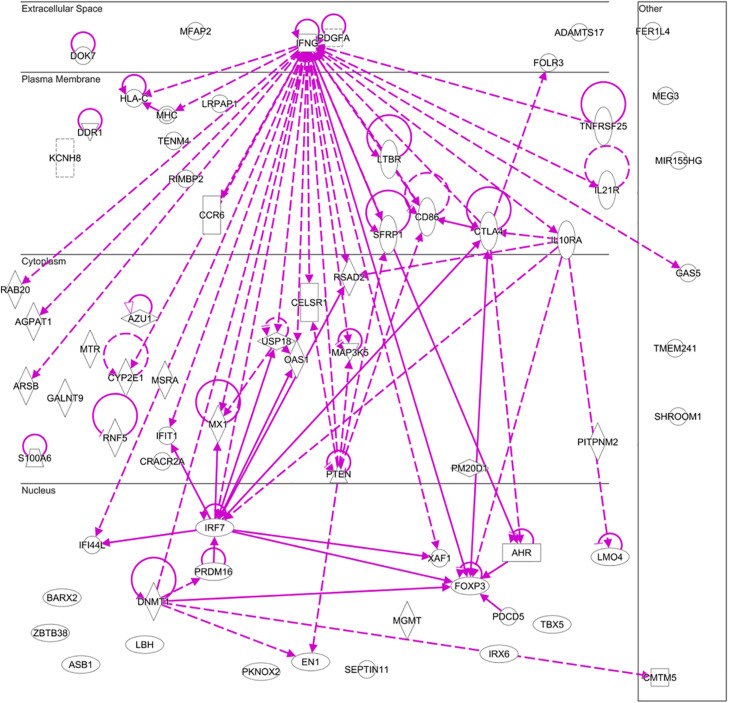
Interaction network constructed by including all differential methylation genes reported in rheumatoid arthritis using an ingenuity pathway analysis (IPA). In this figure, we applied a grow algorithm on the IPA database to connect all the differentially methylated genes with direct and indirect links according to ingenuity core pathway. Gene products were arranged graphically based on the location/function of the associated proteins. Shapes and lines with different styles do not have special representation, but they make different gene productions easy to recognize.

The methylation difference could also explain the different clinical manifestations and mechanisms in different autoimmune diseases. For example, compared with osteoarthritis (OA), 523 low-methylated regions are specific to RA. The regions overlap with specific motifs of transcription factors, such as *GLI1, RUNX2*, and *TFAP2A/C*, which promote the proliferation of synovial cells and the development and migration of plasmacytoid dendritic cells in RA (Ham et al., 2019). In contrast with OA, in which *C18orf45, LMO4, MAP3K5, ODZ4, PKNOX2, SEPT11, MSRA*, and *MIR155HG* are hypomethylated, *PRDM16* is hypermethylated in RA (Glossop et al., 2015). *CCR6*, *CMTM5*, *IL-10R*, *IL-21R*, and *IL-32* are found hypermethylated in SLE and primary Sjögren’s syndrome (pSS), and they are also hypomethylated in RA (Wang et al., 2018). Here, we summarize all the differential methylated genes and construct an interaction network to show the relationship among these differential methylation genes in RA, using the annotations from ingenuity pathway analysis (IPA). We find that these differential methylated genes are not independent, but exhibit an interesting network (Figure 1 and Supplementary Table S1). These studies emphasize that changes in DNA methylation among different autoimmune diseases should be investigated in parallel to identify shared, disease-specific epigenetic dysfunctional elements.

## Epigenetic Regulatory Roles in the Underlying Pathogenesis of Rheumatoid Arthritis

Rheumatoid arthritis fibroblast-like synoviocytes (RA-FLS) are involved in the release of inflammatory mediators and matrix-degrading enzymes, which are key effector cells leading to synovial inflammation and destruction of bone and cartilage (Neumann et al., 2010). The changes in DNA methylation in RA-FLS play important roles in the pathogenesis of RA. Hypomethylation in RA-FLS may be caused by the downregulation of *DNMT1* and *DNMT3A* after inflammatory environmental stimulation (Nakano et al., 2013). Hypomethylation-mediated overexpression of *TBX5* in RA-FLS increases the expression of IL-8, CXCL12, and CCL20, which enhances the inflammatory response in RA (Karouzakis et al., 2014). Hypermethylation of *EBF3* and *IRX1* in RA-FLS mediates the TNF-β pathway and affects the proliferation, apoptosis, and mesenchymal characteristics of RA-FLS (Park et al., 2013). In addition, limb bud and heart development (*LBH*) is a transcription regulator of the cell cycle and is involved in the control of cell growth and proliferation. The activity of *LBH* decreases by enhanced methylation, which may contribute to synovial hyperplasia by initiating the cell cycle (Ekwall et al., 2015; Hammaker et al., 2016).

The critical role of methylation changes in peripheral blood mononuclear cells (PBMCs) during the pathogenesis of RA was also extensively investigated. The demethylation levels in the promoter regions of *IFNG* and *CNS-1* in peripheral blood CD4+ T cells of patients with RA increase, which establishes stable effector/memory during Th1-cell interaction (Dong et al., 2013). In addition, DNA methylation in the promoter region of *CTLA-4* inhibits the activation of the tryptophan-degrading enzyme indoleamine 2,3-dioxygenase pathway, which results in the defunctionalization of Tregs (Cribbs et al., 2014). Further evidence shows that activation of *PRLR* mediates the demethylation of TNF-α in peripheral CD14+ monocytes and increases the release of TNF-α (Tang et al., 2014). Hypomethylation of *ZBTB38* decreases *IL1R2* expression in B cells to interfere with the anti-inflammatory pathway (Ocsko et al., 2018). Hypermethylation of the *AHR* promoter region is associated with the formation of germinal center in the B cells (Toth et al., 2019). Evidence shows that demethylation in the proximal promoter region of *ER* mediates its increased expression in peripheral blood lymphocytes of RA patients, which is found to induce the occurrence of RA (Liu et al., 2014). Hypomethylation of DNA in apoptotic CD4+ T cells upregulates the production of IL-6 in macrophages and downregulates the production of TGF-β in the DCs and B cells (Notley et al., 2017). This evidence demonstrated that DNA methylation plays important roles in the pathogenesis of RA.

## DNA Methylation as a Biomarker for Rheumatoid Arthritis Diagnosis

At present, the serological markers used for the diagnosis of RA are anti-citrullinated protein antibodies (ACPAs) and rheumatoid factor (RF). However, the sensitivity of the test is only about ∼70% (Nishimura et al., 2007). It is urgent to find novel markers to increase the diagnosis accuracy, disease onset prediction, and disease progression. Methylation levels of *SHROOM1* in ERA are substantially increased, which can be applied as an early diagnosis biomarker (Karouzakis et al., 2018). Also, the *MHC* region is found hypomethylated in both PBMCs and the whole blood (van Steenbergen et al., 2014). Furthermore, *CD86*, *RAB20*, *XAF1*, *FOLR3*, *LTBR*, *KCNH8*, *DOK7*, *PDGFA*, *PITPNM2*, and *CELSR1* in B cells show significant methylation differences in RA (Tseng et al., 2019b). In another study, *S100A6* and *EFCAB4B* promoter regions in the whole blood and *IFN*-related genes (*IFIT1*, *IRF7*, *MX1*, *OAS1*, *USP18*, *RSAD2*, *IFI44L*) in CD4+ T cells are hypomethylated, indicating these methylation signals might be used as biomarkers for RA diagnosis (Svendsen et al., 2016; Chen et al., 2019). *AZU1*, *LTBR*, and *RTEL1* are hypomethylated and involved in the autoimmune signaling cascade, indicating potential roles as epigenetic susceptibility markers (Wang et al., 2018). Hypomethylation of *CYP2E1* is associated with disease activity and can be used as a disease activity marker (Mok et al., 2018). *CD1C, TNFSF10, C6ORF10*, and *UBASH3A* have the potential to be used as RA risk markers (Julia et al., 2017; Anaparti et al., 2019; Guderud et al., 2020). Finally, except gene-based methylation biomarkers, some other pathway or genome-wide indicators also show interesting biomarker performances. For example, a recent study showed that methylation-derived neutrophil-to-lymphocyte ratio (mNLR), detected from peripheral blood DNA, is increased during RA onset (Ambatipudi et al., 2018). Overall, all these pieces of evidence demonstrate that DNA methylation could be used as a robust biomarker for disease risk assessment and progression, and we expect more future advancements in this field.

## Epigenetic Modules Mediated by Methylation are Promising RA Drug Targets

Epigenetic modifications are dynamically variable, and their reversibility is an attractive characteristic to develop new drugs. RA is highly related to the functional defects of Tregs, and *FOXP3* stabilizes the immune regulatory function of Tregs. A recent study showed that daurinol induces hypomethylation of *FOXP3*, promotes Tregs differentiation and stabilization, inhibits Th17 differentiation through the Nrp1-Pten-Akt-Foxp3 signaling pathway, and alleviates the severity of RA (Park et al., 2019). In another study, expansion of Tregs *in vitro* with rapamycin was shown to improve the function and stability of Tregs, which maintain *FOXP3* high expression and strong inhibitory ability, in which *TNFR2* maintains *FOXP3* expression by restricting DNA methylation (Rossetti et al., 2015). These pieces of evidence indicate hypomethylation of *FOXP3*, which may present an interesting drug target for the treatment of RA. Meanwhile, evidence shows that methotrexate (MTX) can restore Treg inhibition through the demethylation of *FOXP3* upstream enhancer and increases the expression of *FOXP3* and *CTLA-4*, providing a new mechanism for MTX (Cribbs et al., 2014). MTX can also reverse DNA hypomethylation in T cells, B cells, and monocytes in patients with RA (de Andres et al., 2015). Furthermore, patients with higher baseline global DNA methylation levels in RA exhibit lower MTX response (Gosselt et al., 2019). MTX can also reduce *MTR* expression in rheumatoid nodules, affecting remethylation mediated by *MTR* and *MTRR*, indicating the important roles of DNA methylation in RA pathogenesis and drug response mechanism (Houlder et al., 2017). Recently, methylation inhibitors (5-Azadc) showed the ability to upregulate *PTEN*; downregulate *HOTTIP*; weaken the enrichment of *DNMT3B* in the *SFRP1* promoter region; and downregulate the expression of β-catenin, TNF-a, IL-6, IL-1β, and CCL-2 (Miao et al., 2013; Li X.F. et al., 2019; Hu et al., 2020). These changes further regulate the AKT and Wnt signaling pathways to aid the remission of RA. *SFRP4* is a negative regulator of the Wnt signaling pathway. Evidence shows that *miR-152* indirectly upregulates *SFRP4* by decreasing the expression of DNMT1 and reduces FLS proliferation (Miao et al., 2014).

The immunomodulatory role of etanercept and adalimumab in mononuclear cells is demonstrated by downregulating the expression of methyltransferase and trimethylation of H3K4, H3K27, H3K36, and H3K79 at the *CCL2* promoter (Lin et al., 2017). Daphnetin reduces the expression of DNMT1, DNMT3A, and DNMT3B in collagen-induced arthritis rat synovial cells, leading to demethylation of pro-apoptotic genes *DR3*, *PDCD5*, *FasL*, and *p53* and increasing the expression of pro-apoptotic genes (Shu et al., 2014). The combination of *SSAT1* inhibitor DA and methyl donor S-adenosyl methionine can significantly improve overall DNA hypomethylation status in RA-FLS and reduce the adhesion of RA-FLS (Neidhart et al., 2014). Another interesting study shows hypermethylation of the promoters *FER1L4*, *GAS5*, and *MEG3*, leading to their downregulation, which promotes the release of pro-inflammatory factors, contributing to the pathogenesis of RA. Therefore, hypomethylation recovery of *FER1L4*, *GAS5*, and *MEG3* may be a potential therapeutic target for RA (Liu et al., 2019; Yu et al., 2019; Li et al., 2020). Finally, DNA methylation of *LBH*, *CASP8*, *OLIG3*, *IRF5*, *HLA-G*, *ELMO1*, *TRHDE*, *SLCO1C1*, *PLD4*, *AIRE*, and *HLA-DQA1* is involved in the pathogenesis of RA and is recommended as a promising novel therapeutic target (Fan et al., 2016).

In addition, methylation also provides novel biomarkers to predict drug responses. There are four CpGs within exon seven of lipoprotein receptor-associated protein 1 (*LRPAP1*) that are more methylated in non-responders than in good responders, which can be used as a response marker of etanercept (TNF-alpha inhibitor) treatment (Plant et al., 2016). Disease-modifying antirheumatic drug (DMARD) reverses the hypomethylation of *RNF5* and *AGPAT1* promoter regions induced by smoking in patients with RA; therefore, it could be considered as a therapeutic target for RA (Svendsen et al., 2016). Meanwhile, two methylation loci (cg03018489 and cg14345882) are significantly correlated with DMARD treatment response (Glossop et al., 2017). In the good responders to MTX, methylation levels of cg23700278, cg27427581, cg04334751, and cg26764200 increase significantly after MTX treatment (Nair et al., 2019). In summary, the drugs that improve the abnormal methylation could be promising for the treatment of RA, and the genes involved in the epigenetic regulation of RA could be considered as novel drug targets for pharmaceutical companies and to explain the novel mechanism of drug action. Meanwhile, the differential DNA methylation can be used as a promising biomarker to predict the drug responses.

## The Advancement of Histone Methylation Research in Rheumatoid Arthritis

Histone methylation is also involved in the pathogenesis of RA. Histone H3 trimethylated at lysine 4 (H3K4me3) in SF is associated with the opening of arthritis-activated chromatin, making the promoters of pathogenic genes highly active to drive transcription (Ntougkos et al., 2017). A previous study shows that H3K4me3 is increased in the promoter region of *MMP*, which is positively correlated with the expression of MMP-1, MMP-3, MMP-9, and MMP-13 in RA-FLS (Araki et al., 2016). Peptidylarginine deiminase type-4 (PADI4) inhibits *p21* transcription by modifying histone H3 arginine at the *p21* promoter region, which protects FLS from apoptosis and promotes the pathogenesis of RA (Fan et al., 2017). The expression of Jumonji domain-containing protein 3 (JMJD3) in RA-FLS is upregulated. JMJD3 specifically demethylates trimethylated lysine, which is directly involved in the activation of *TLR2* through the demethylation of H3K27me3 promoter and promotes RA inflammation (Wu et al., 2019). Aiming at the pathogenesis of RA caused by abnormal histone methylation is useful in improving the inflammation in RA. For example, the application of JMJD3 inhibitor, GSK-J4, inhibits the methylation of H3K27me3 at the *TLR2* promoter, significantly relieving the destruction and inflammation of articular cartilage (Wu et al., 2019). Therefore, histone modification regulators are potential and promising drug targets for RA therapy and drug development.

## Challenges and Opportunities of the Current Epigenetics Research in Rheumatoid Arthritis

Research on epigenetics highlights the role of methylation changes in the pathogenesis, diagnosis, treatment, and prognosis of RA. DNA and histone methylation, including *TBX5* (Karouzakis et al., 2014), *FOXP3* (Kennedy et al., 2014), *AHR* (Toth et al., 2019), *HKMT* (Araki et al., 2018), and H3K4me3 (Araki et al., 2016), affect the proliferation, migration, apoptosis, and inflammation of immune cells, which explains the pathogenesis of RA. Preclinical differential methylation changes, such as the mNLR, contribute to early diagnosis of RA (Ambatipudi et al., 2018). The identified differential methylation genes can be applied as useful biomarkers to predict RA progression and disease severity and provide potential therapeutic targets for RA. Epigenetic modifications as drug targets could provide a new direction of pharmacological research for the development of novel drugs that alleviate clinical pressures of high toxicity, low efficiency, and high cost of the current medicine. For example, demethylation of *FOXP3* is used as a biomarker to evaluate the therapeutic drug response, which provides a direction for the precision treatment of RA (Tabares et al., 2018). *MALAT1* promotes *CTNNB1* promoter methylation and inhibits the Wnt signaling pathway, and has been shown as an interesting potential therapeutic target for RA (Li G.Q. et al., 2019). In addition, DNA and histone methylation in individuals may change the response to drugs; identifying the drug response markers may be helpful for personalized medication development.

Although DNA methylation and histone methylation research have shown significant progress in RA, the current research presents several severe concerns. For example, there is no comprehensive study to investigate the relationship between differential methylation and disease severity and inflammation indicators. Future research should also strive to extended cohort studies to improve statistical robustness. Studies of differential methylation and pathways have examined the corresponding sites, but few functional tests have been performed. Different experimental approaches should be integrated to verify the results and obtain a solid conclusion with a large sample size. Moreover, further studies are needed to reveal the functions and targets of differential methylation and illuminate RA pathogenesis and drug discovery. We expect multi-omics analyses to be conducted to illustrate the roles of the interactions between epigenetic elements (5 mC and histone modification) in multiple autoimmune diseases as well as the interaction between genetics and epigenetics for a better understanding of RA pathogenesis.

## Author Contributions

SG and DH conceived of content. SG, LX, and CC drafted the review, which was edited by RZ and YJ. All authors contributed to the article and approved the submitted version.

## Conflict of Interest

The authors declare that the research was conducted in the absence of any commercial or financial relationships that could be construed as a potential conflict of interest.

## Supplementary Material

TABLE S1 Interaction network among rheumatoid arthritis differential methylation genes.
